# In silico evaluation of the role of PEA3 subfamily ETS transcription factors in chemoresistance in ovarian cancer

**DOI:** 10.55730/1300-0144.6223

**Published:** 2026-04-21

**Authors:** Fevzi Coşkun SÖKMEN, Laika KARDANA, Hikmet YILMAZ, Ahmet Çağlar ÖZKETEN, Aynur KARADAĞ GÜREL, Özge ATAY, Hasan Hüseyin KAZAN

**Affiliations:** 1 Department of General Internal Medicine, Gülhane Faculty of Medicine, University of Health Sciences, Ankara, Turkiye; 2 Department of Medical Biology, Gülhane Faculty of Medicine, University of Health Sciences, Ankara, Turkiye; 3 DESAM Research Institute, Near East University, Mersin 10, Turkiye; 4 Department of Biochemistry, Middle East Technical University, Ankara, Turkiye

**Keywords:** Ovarian cancer, cisplatin resistance, ETS gene family, ETV4, CIC

## Abstract

**Background/aim:**

The E26 transformation-specific family of transcription factors regulates the cell cycle and apoptosis that are crucial for carcinogenesis. More specifically, the PEA3 subfamily—comprising ETS variant (ETV) transcription factors ETV1, ETV4, and ETV5—has been implicated in multiple oncogenic signaling pathways and chemotherapy resistance. While cisplatin is still a widely used chemotherapeutic treatment for ovarian cancer, its therapeutic efficacy is sometimes limited by the induction of resistance mechanisms. The present study investigates the potential role of PEA3 subfamily genes in cisplatin resistance in ovarian cancer through comprehensive in silico analyses.

**Materials and methods:**

Cisplatin response data for ovarian cancer were obtained from the Cancer Treatment Response Database version 2 (CTR-DB2), and the relevant dataset was analyzed. Candidate genes, including members of the PEA3 transcription factor subfamily, were analyzed with a multigene biomarker model, and a receiver operating characteristic analysis was used to measure predictive ability. GEPIA2, KMplotter, and cBioPortal were used for survival analysis, while the TNMplot, cBioPortal, and Clinical Proteomic Tumor Analysis Consortium datasets were used for multiomics characterization and differential expression.

**Results:**

The ETV4–CIC gene pair in the CTR-DB2 validation dataset achieved the highest predictive performance for cisplatin response in ovarian cancer with an area-under-curve of 0.939, clearly separating responders from nonresponders. No other gene combinations outperformed this model, demonstrating only moderate predictive capacity. The ETV4–CIC gene pair was also associated with disease-free survival. Differential expression analysis showed downregulation of CIC and upregulation of ETV4 in tumor tissues. Genomic and proteomic analyses confirmed alterations in both genes, while correlation analysis suggested complementary biological roles.

**Conclusions:**

The ETV4–CIC gene pair effectively discriminated between cisplatin responders and nonresponders in ovarian cancer. Accordingly, the ETV4–CIC signature may serve as a useful biomarker for cisplatin response prediction and patient stratification in precision oncology.

## 1. Introduction

The E26 transformation-specific (ETS) family of transcription factors play a critical role in both physiological and pathophysiological conditions [1]. Mechanistically, members of the ETS family participate in cell cycle and cell death pathways, underlying their association with tumorigenesis [2,3]. Within the ETS family, the PEA3 subfamily—including ETS variant transcription factor 1 (ETV1), ETV4, and ETV5—is of particular interest due to its involvement in multiple pathways, including cell growth, invasion, and migration as key regulators. These transcription factors have been reported to be oncogenic through their roles in cell cycle regulation, epithelial-mesenchymal transition (EMT), apoptosis, and chemoresistance [3].

Chemoresistance in particular is a major challenge in cancer therapy due to its association with treatment failure and cancer recurrence, driven by complex transcriptional and signaling alterations [4,5]. Among the available chemotherapeutic agents, cis-diamminedichloroplatinum (cisplatin) is widely used in the treatment of ovarian cancer [6]. Despite its therapeutic efficacy, cisplatin-based chemotherapy often results in chemoresistance and treatment failure [7]. A deeper understanding of the molecular mechanisms is therefore important for the development of effective treatments to overcome cisplatin resistance [8,9]. ETV1, ETV4, and ETV5 are regulated by such major oncogenic signaling pathways as mitogen-activated protein kinase 1 (MAPK) and PI3K/Akt. However, the inhibition of these pathways during cancer therapy may lead to the reactivation of PEA3 subfamily genes, contributing to drug resistance [3]. It has been suggested that ETV4 may be a crucial target for counteracting chemoresistance in ovarian cancer [10]. ETV4 and ETV5 expression levels in ovarian cancer can be regulated by the miR-1307/Capicua (CIC) signaling pathway. Mechanistically, miR-1307 targets the 3′ untranslated region (UTR) of the CIC gene, the downregulation of which allows ETV4 and ETV5 to be upregulated. Dysregulation of this axis promotes chemoresistance in ovarian cancer by increasing ETV4 and ETV5 expression and multidrug resistance protein 1 (MDR1) activity [11]. Nevertheless, such efforts emphasizing the possible roles of those transcription factors in cancer drug resistance require further molecular and/or in silico studies.

The present study investigates the potential role of PEA3 subfamily genes (ETV1, ETV4, and ETV5) of the ETS transcription factors in chemotherapeutic resistance, particularly to cisplatin in ovarian cancer, using in silico analyses. To this end, the study evaluates the potential of PEA3 subfamily genes as biomarkers for distinguishing cisplatin responders from nonresponders. Our results may contribute to the development of therapeutic strategies for ovarian cancer.

## 2. Materials and methods

### 2.1. Data source and study design

Drug response data were sourced from the publicly accessible Cancer Treatment Response Database version 2 (CTR-DB2) [12]. A comprehensive search of the database was conducted to identify datasets related to cisplatin-treated ovarian cancer. Since only one dataset (CTR_Microarray_30-I) met the inclusion criteria, this dataset alone was used for subsequent analyses. Of the patients detailed in the dataset, 10 were treatment-resistant whereas 18 responded to cisplatin either completely or partially.

### 2.2. Biomarker discovery and ROC analysis

PEA3 transcription factor genes along with other candidate genes were evaluated as possible biomarkers through a multigene model. The discrimination of cisplatin responders from nonresponders was achieved through the analysis of several gene combinations. Receiver operating characteristic (ROC) analysis was used to evaluate predictive performance, and combinations with an area under the curve (AUC) above 0.90 were selected for further analysis.

### 2.3. Survival analyses

The clinical relevance of the selected gene signature was first evaluated using GEPIA2 through disease-free survival (DFS) and overall survival (OS) analyses with log-rank tests [13]. The findings were independently validated using KMplotter, and the patients were subsequently classified into high- and low-expression groups based on their combined expression scores [14]. The robustness of the results was evaluated by calculating false discovery rate (FDR) values. cBioPortal was used for additional survival analyses, including disease-specific survival (DSS) and genomic context [15].

### 2.4. Expression analysis and multiomics characterization

Differential gene expression between tumor and normal tissues were assessed using TNMplot [16]. cBioPortal was used for the analysis of genomic cooccurrence, coexpression, and cancer type summaries [15]. mRNA expression z-scores relative to diploid samples were used to minimize any bias associated with copy number variations. Protein expression was further validated based on mass spectrometry data from the Clinical Proteomic Tumor Analysis Consortium (CPTAC) [17]. In addition, coexpression and OncoPrint analyses were conducted to determine whether the genes provide complementary or redundant information. Finally, recent multiomics studies in ovarian cancer and chemotherapy resistance were reviewed to support the interpretation of our results and underscore the significance of multilayer biomarker discovery approaches [17,18].

## 3. Results

**Figure 1:**
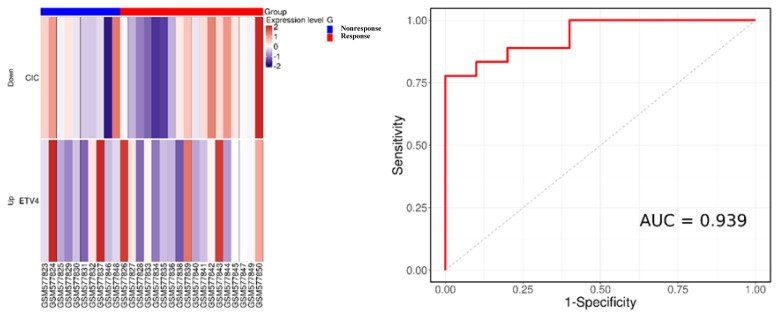
ROC curves and heatmap visualization from CTR-DB2 showing the predictive performance of biomarker combinations. The ETV4–CIC pair achieved the highest AUC (0.939), with clear separation between responders and nonresponders.

Among the tested gene pairs, the ETV4–CIC signature achieved the highest predictive performance (AUC = 0.939) in the validation analyses of combined biomarkers using CTR-DB2. In contrast, ETV1–ETV4 (AUC = 0.767), ETV1–ETV5 (AUC = 0.650), ETV1–CIC (AUC = 0.728), ETV4–ETV5 (AUC = 0.806), and ETV5–CIC (AUC = 0.772) demonstrated moderate predictive performance. Several other gene signatures (ETV1–ETV4–ETV5: AUC = 0.783; ETV1–ETV4–CIC: AUC = 0.878; ETV1–ETV5–CIC: AUC = 0.717; ETV4–ETV5–CIC: AUC = 0.883; ETV1–ETV4–ETV5–CIC: AUC = 0.878) failed to exceed the performance of the ETV4–CIC pair. The discriminative capacity of the ETV4–CIC pair was further supported by the heatmap generated from the CTR-DB2 dataset, demonstrating clear separation between responders and nonresponders (Figure 1). Notably, none of the gene combinations reached high AUC values for cisplatin resistance in other cancer types (n = 744) or for resistance to other drugs in ovarian cancer (n = 486), suggesting that the ETV4–CIC signature may be specific to ovarian cancer and cisplatin resistance.

**Figure 2:**
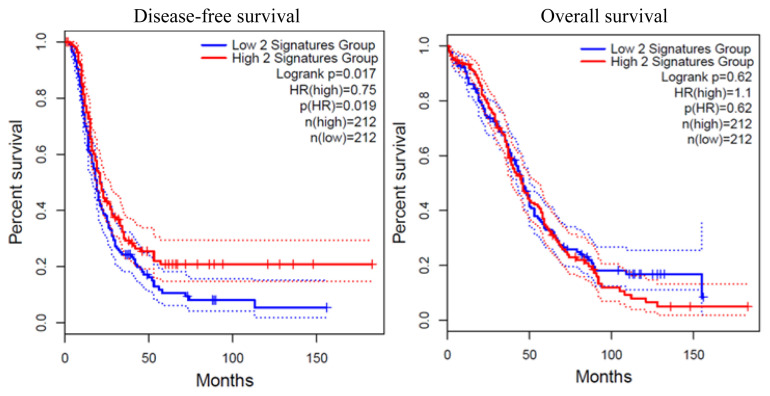
Kaplan–Meier plots generated with GEPIA2 demonstrating the significant association of the ETV4–CIC signature with DFS (p = 0.017) but no significant difference in OS (p = 0.62).

Survival analysis performed using GEPIA2 revealed a significant association between the combined expression signature and DFS (p = 0.017) whereas no significant difference was observed for OS (p = 0.62; Figure 2).

**Figure 3:**
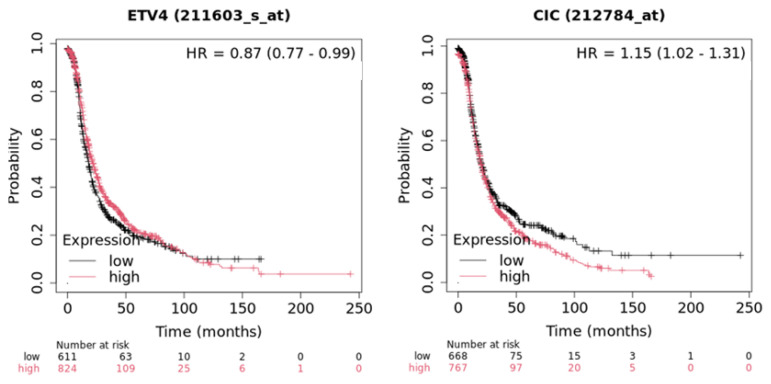
KMplotter validation of survival outcomes across independent datasets. Statistical analysis was performed on ETV4 (p = 0.033) and CIC (p = 0.028) across multiple datasets (FDR > 50%).

Validation using KMplotter demonstrated a modest prognostic effect, as patients with high expression levels had longer survival (22 vs 18 months; p = 0.033). However, the FDR exceeded 50%, reducing the statistical robustness of the result. Consistent with this trend, Kaplan–Meier analyses across multiple datasets showed longer DFS in the high ETV4–CIC group, while no significant differences were identified for OS, progression-free survival (PFS), or DSS, all of which had log-rank p-values over 0.3 (Figure 3).

TNMplot-based differential expression analysis demonstrated that CIC was markedly downregulated while ETV4 was upregulated in tumor samples compared to normal tissues. Moreover, the biological relevance of the findings was supported by comparisons between metastatic vs nonmetastatic tumors. For ETV4, the fold change of tumor vs normal was 5.58 whereas that of metastasis vs nonmetastasis was 0.24, and for CIC, these values were 1.40 and 0.80 for tumor vs normal and metastasis vs nonmetastasis, respectively.

Genomic profiling with cBioPortal OncoPrint revealed that the CIC and ETV4 alterations included mutations, copy-number changes, and abnormal mRNA expression. Importantly, CIC depletion and ETV4 upregulation were frequently observed together, indicating a functional interaction between the two genes. Normalized mRNA z-scores reduced any bias associated with copy number variations. Consistent with these findings, proteomic validation using CPTAC data supported the results at the protein level, revealing increased ETV4 and decreased CIC expression in some ovarian cancer samples.

**Figure 4:**
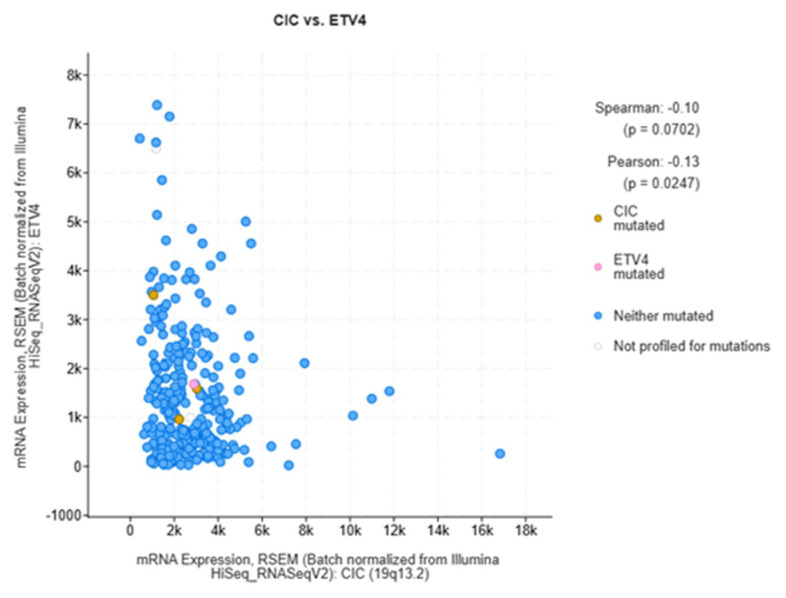
Scatterplots of CIC vs ETV4 expression across ovarian cancer datasets.

Correlation analysis using cBioPortal demonstrated a weak but statistically significant negative correlation between CIC and ETV4 expression (Pearson r = −0.13, p = 0.0247; Spearman r = −0.10, p = 0.0702; Figure 4), indicating that they have complementary roles.

## 4. Discussion

In the present study, downregulation of CIC accompanied by upregulation of ETV4 was identified as a potential combined biomarker for predicting cisplatin responsiveness in ovarian cancer. Validation in the CTR-DB2 cohort confirmed a strong predictive performance (AUC = 0.939), emphasizing the clinical ability of this two-gene signature to effectively distinguish between responders and nonresponders.

Ovarian cancer is a potentially fatal gynecological malignancy with global relevance. Cisplatin is one of the leading treatment agents worldwide; however, its efficacy can be affected by both genetic and epigenetic mechanisms that contribute to treatment resistance [19]. Cisplatin primarily binds to DNA, inducing DNA damage that leads ultimately to cell death, while also triggering diverse cytotoxic mechanisms such as the production of reactive oxygen species (ROS) and the modulation of apoptosis-related pathways [20]. Cisplatin resistance may also occur through its direct interactions with certain compounds and the dysregulated expression of critical genes [21]. Understanding these altered expression patterns is crucial for overcoming cancer drug resistance [5,22].

**Figure 5:**
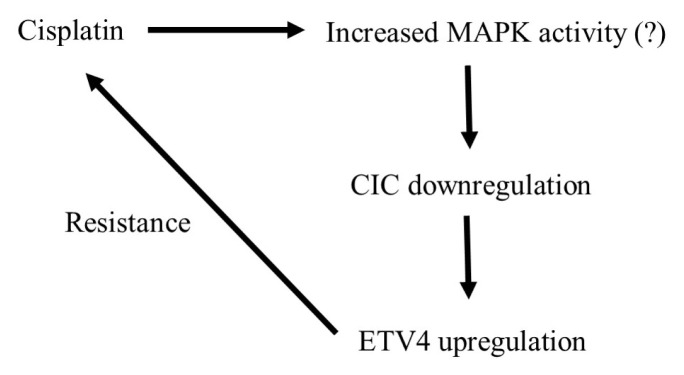
Proposed mechanisms for cisplatin resistance associated with the ETV4–CIC signature in ovarian cancer.

In cancer drug resistance, dysregulation of apoptosis highlights the critical role of transcription regulation and function. In this context, the PEA3 subfamily of ETS transcription factors should be emphasized due to the upregulation of the ETV1, ETV4, and ETV5 family members—regarded as oncogenic transcription factors owing to association with cell cycle, apoptosis, cell migration, and EMT—in diverse cancers [3]. Ovarian cancer is one pathophysiological condition in which the PEA3 subfamily has been widely studied [23–25]. These transcription factors are transcriptionally regulated by CIC, a repressor controlled by the MAPK signaling pathway [3]. Importantly, cisplatin activates the MAPK pathway which suppresses CIC, thereby upregulating the PEA3 subfamily members and promoting carcinogenesis and drug resistance [3,26]. Our results indicate that CIC is downregulated while ETV4 is upregulated in cisplatin resistant ovarian cancer, further supporting this mechanism (Figure 5). Upregulation of ETV4 may further upregulate MDR1, which plays a critical role in cisplatin resistance [3,11].

In addition to the resistance phenotype, DFS was evaluated using GEPIA2 and KMplotter, revealing that the ETV4–CIC signature had significant prognostic value. High ETV4–CIC scores were associated with a decreased likelihood of tumor recurrence, while no significant association was observed between ETV4–CIC signature and OS (GEPIA2 OS p = 0.62; KMplotter FDR >50%), suggesting a limited capacity to predict long-term outcomes. As discussed previously, the differences in statistical significance between DFS and OS outcomes could be explained by the clinical and demographic variations between patients [27].

Expression analysis performed using TNMplot provided biological validation, demonstrating consistent downregulation of CIC and upregulation of ETV4 in tumor tissues compared with normal samples, as well as between the responder and nonresponder groups. Comparisons of mutant and wild-type cases further supported the biological relevance of these alterations. Genomic profiling with cBioPortal OncoPrint revealed that alterations in CIC and ETV4 included mutations, copy-number changes, and mRNA dysregulation. The frequent coocurrences of CIC loss and ETV4 recorded suggested functional interplay. Normalized mRNA z-scores reduced the copy-number bias, and proteomic validation using CPTAC confirmed these findings at the protein level, with ETV4 protein upregulation and CIC protein reduction observed in subsets of ovarian cancer samples. Correlation analysis using cBioPortal revealed a weak but statistically significant negative correlation between CIC and ETV4 expression (Pearson r = −0.13, p = 0.0247; Spearman r = −0.10, p = 0.0702), indicating that these genes act in complementary rather than redundant pathways. This observation is supported by previous studies reporting that multigene models provide greater predictive accuracy than single-gene predictors by integrating heterogeneous biological mechanisms associated with chemoresistance [3,17,18].

Although the gene signature has shown potential prognostic value for predicting recurrence, its limited impact on overall survival suggests that additional omics approaches and the inclusion of more genes to improve prognostic accuracy are required. Incorporating metabolomics, epigenomics, and single-cell transcriptomics, together with clinical variables, may enhance the utility of the gene signature in guiding therapeutic decisions.

Despite its direct findings, the present study has several limitations. To begin with, there was only one dataset available (CTR_Microarray_30-I) related to cisplatin-treated ovarian cancer in CTR-DB2, and the small sample size (n = 28) was another limitation. Additionally, the KMplotter analyses yielded FDR values above 50%, indicating limited prognostic robustness. Moreover, tumor stage, histological subtype, disease status, and treatment durations/dosages were not documented, which may misdirect the findings of the study.

## 5. Conclusion

This study presents a multigene biomarker model that was developed for the accurate prediction of cisplatin responsiveness in ovarian cancer. The potential discriminative capacity of ETV4–CIC was further supported by a heatmap and ROC evaluations using the CTR-DB2 validation cohort, supporting its significance as a biomarker. Survival analyses conducted using GEPIA2 and KMplotter revealed significant prognostic relevance for disease-free survival, whereas no meaningful association was observed with overall survival. TNMplot-based differential expression analysis demonstrated decreased CIC levels and increased ETV4 levels. Genomic profiling using cBioPortal OncoPrint revealed that CIC and ETV4 alterations included mutations, copy-number changes, and mRNA dysregulation. Collectively, these results may highlight the translational potential of CIC and ETV4 as a clinically significant biomarker duo for patient classification in precision oncology.
